# Efficacy of Combined Endoscopic Lithotomy and Extracorporeal Shock Wave Lithotripsy, and Additional Electrohydraulic Lithotripsy Using the SpyGlass Direct Visualization System or X-Ray Guided EHL as Needed, for Pancreatic Lithiasis

**DOI:** 10.1155/2014/732781

**Published:** 2014-06-05

**Authors:** Ken Ito, Yoshinori Igarashi, Naoki Okano, Takahiko Mimura, Yui Kishimoto, Seiichi Hara, Kensuke Takuma

**Affiliations:** Division of Gastroenterology and Hepatology, Toho University, Omori Medical Center, 6-11-1 Omori-nishi, Ota-ku, Tokyo 143-8541, Japan

## Abstract

*Introduction*. To evaluate the efficacy of combined endoscopic lithotomy and extracorporeal shock wave lithotripsy (ESWL), and additional electrohydraulic lithotripsy (EHL) as needed, for the treatment of pancreatic duct stones, we retrospectively evaluated 98 patients with chronic pancreatitis and pancreatic lithiasis. *Methods*. For the management of main pancreatic duct (MPD) stones in 98 patients, we performed combined endoscopic treatment (ET)/ESWL therapy as the first treatment option. When combined ET/ESWL was unsuccessful, EHL with the SpyGlass Direct Visualization system or X-ray guided EHL was performed. Outpatient ESWL was reserved as one of the final treatment options. *Results*. Fragmentation was successful in 80 (81.6%) patients as follows: combined ET/ESWL: 67 cases; SpyGlass EHL: 4 cases; X-ray guided EHL: 3 cases; and outpatient ESWL: 6 cases. Successful outcome was obtained by combined ET/ESWL in 67 of the 98 patients (74.5%), by EHL in 7 of 14 patients (7.1%), and by outpatient ESWL in 6 of 6 patients (6.1%). Negotiating the guidewire through a severe MPD stricture was significantly associated with a higher rate of stone fragmentation (*P* = 0.0003). *Conclusions*. In cases where combined ET/ESWL was not successful for stone clearance, EHL using the SpyGlass system or X-ray guided EHL was effective in cases where the guidewire could be negotiated through the MPD stricture and it increased the fragmentation rate.

## 1. Introduction

Pancreatic lithiasis in chronic pancreatitis, especially in the main pancreatic duct (MPD), may cause pain due to pancreatic stasis or increased MPD pressure. Pancreatic stone elimination is a suitable treatment for pain removal and prevents acute exacerbation of pancreatitis [1]. Extracorporeal shock wave lithotripsy (ESWL) is typically the first treatment option in Japan, because it is minimally invasive and has fewer early complications than other treatments [2]. Complications of ESWL include acute obstructive pancreatitis due to stone lithotripsy, and patients should therefore be admitted for postmonitoring of ESWL and for preventing complications requiring endoscopic pancreatic sphincterotomy (EPST) [3]. Management in cases of large-diameter stones requires lithotripsy, for which combined endoscopic treatment (ET)/ESWL therapy is more effective than ESWL therapy alone [4]. However, in cases where such combination therapy is unsuccessful, surgical or similar intervention is typically required for symptomatic patients [5]. Electrohydraulic lithotripsy (EHL) is one such intervention that has been shown to be efficacious [6]. In addition, an outpatient ESWL approach can be helpful in shortening hospital stays for those patients whose pain can be temporarily relieved, who cannot afford a long hospital stay due to financial or work-related reasons, or whose stone has been incompletely fragmented by ESWL. Accordingly, our department performs EHL as a second treatment option, and, in the event that EHL is unsuccessful, outpatient ESWL is performed in cases of temporary pain relief or radiolucent stones. In this study, to evaluate the efficacy of combined endoscopic lithotomy and ESWL, and additional EHL as needed, for the treatment of pancreatic duct stones, we retrospectively evaluated cases of symptomatic pancreatic duct stones treated at our institution.

## 2. Indications, Patients, and Methods

**Figure 1:**
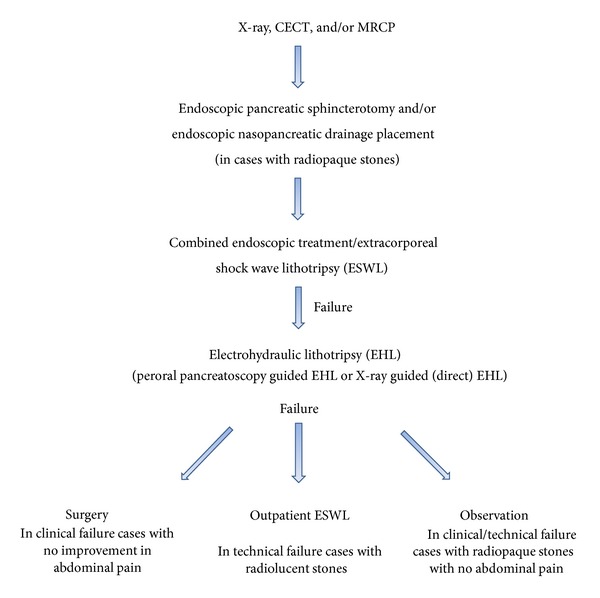
Management flow of pancreatic lithiasis.

The management flow of chronic pancreatic lithiasis performed at our center during the study period is shown in Figure 1. Indication for chronic pancreatic lithiasis treatment was defined as “absolute” or “relative,” as shown in the following.

*Treatment Indications Are as Follows*

1. Absolute indication ((a) + (b)):
  - presence of abdominal symptoms,
  - presence of pancreatic duct stone in the Santorini duct or Wirsung duct, and upstream MPD dilatation detected by diagnostic imaging (CECT, MRCP).
2. Relative indication:
  - no abdominal symptoms in patients with diabetic mellitus and exacerbation of glucose tolerance on diagnostic imaging with 1(b).

All patients underwent X-ray, contrast-enhanced computed tomography (CECT), and/or magnetic resonance cholangiopancreatography (MRCP) before treatment to distinguish radiolucent from radiopaque stones. Standard endoscopic retrograde cholangiopancreatography (ERCP) was performed before treating the pancreatic duct stones in all cases, and ESWL was considered in cases of endoscopically unremovable stones.

**Figure 2:**
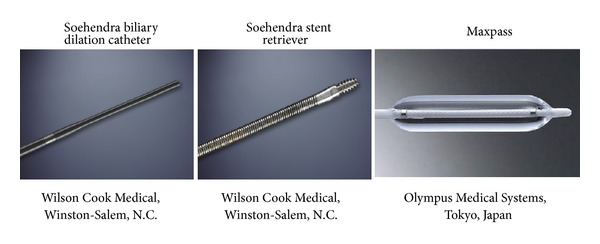
Dilators for MPD stricture.

All procedures were performed with a TJF240 or TJF260V duodenoscope (Olympus Medical Systems, Tokyo, Japan). EPST was always performed as the first step, and when selective intubation was difficult, precutting was performed with EPST as a secondary procedure [7]. When pancreatic duct stricture was recognized on pancreatography, a guidewire was negotiated through the tail of the pancreatic duct as close as possible to the tail of the MPD, and dilatation was attempted. Although we typically used 0.035-inch Revowave standard type and Revowave hard-type guidewires (Piolax Medical Devices, Inc. Kanagawa, Japan), we used a 0.025-inch Visi-Glide guidewire (Olympus Medical Systems) when stricture was severe because traversing the stricture effectively with a standard guidewire is difficult. When the guidewire was negotiated through the tail of the MPD, a dilatation device such as a Soehendra biliary dilatation catheter (SBDC; Wilson Cook Medical, Winston-Salem, NC), Soehendra stent retriever catheter (SSR; Wilson Cook Medical), or Maxpass (Olympus Medical Systems) was used (Figure 2). In cases of radiopaque stones, an endoscopic nasopancreatic drain (ENPD) was placed, and contrast media was infused through the ENPD during ESWL to identify stones. To improve the efficacy of lithotripsy, a slow shock wave (45 pulses/min) was applied using an electromagnetic Siemens Lithoskop (Siemens AG, Munich, Germany).

**Figure 3:**
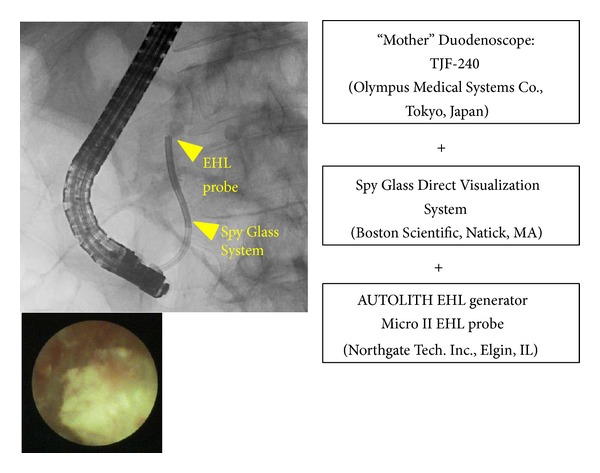
Peroral pancreatoscopy guided EHL using the SpyGlass Direct Visualization system.

**Figure 4:**
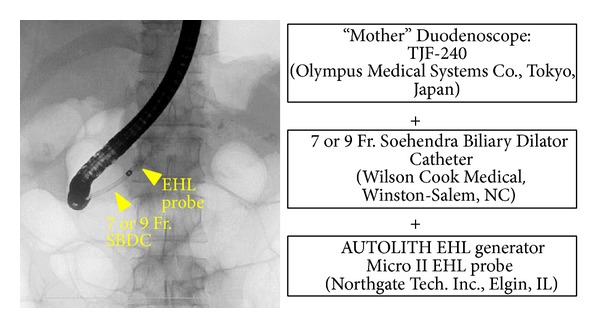
X-ray guided EHL.

If the endoscopic lithotomy/ESWL combination was unsuccessful, EHL was performed as a second attempt. Before 2010, we used a 3.5 mm diameter CHF TYPE BP 260 baby scope (Olympus Medical Systems) for EHL. However, because of its naïve characteristics and fragility, we switched to the 10 Fr SpyGlass Direct Visualization system (Boston Scientific, Natick, MA) for EHL. The NORTECH MICRO II 1.9 Fr 250 cm EHL Probe (Northgate Technologies Inc., Elgin, IL) and NORTECH AUTOLITH EHL Generator (Northgate Technologies Inc.) were optimized for use with the SpyGlass Direct Visualization system (Figure 3). Alternatively, X-ray guided EHL using a 7 Fr biliary dilator as an outer sheath was performed when a 10 Fr SpyGlass system delivery catheter was difficult to insert into the MPD stricture (Figure 4).

Stone location in the MPD was defined as head or body/tail. The number of stones in the MPD was defined as single or multiple. MPD stricture was defined as stricture or severe stricture (requiring the use of a SSR or SBDC).

Analysis was conducted to determine the outcomes of our management flow: combined ET/ESWL as the first option, peroral pancreatography (POPS) guided EHL or X-ray guided EHL as the second option, and continuing outpatient ESWL as the third option when the previous treatments had failed. Clinical success (improvement in abdominal complaints) and technical success (clearance of target pancreatic stone) and the efficacy of POPS guided EHL and direct EHL were evaluated as follows. 

*Definitions for Clinical and Technical Success*

*Clinical Success.* Clinical success is defined as improvement in abdominal symptoms (e.g., abdominal pain, back pain, and abdominal discomfort) after EPST/precutting and/or pancreatic lithiasis treatment.

*Technical Success.* Technical success is defined as clearance of the target pancreatic stone after the treatment (e.g., endoscopic treatment/ESWL/EHL).

In addition, using multiple logistic regression analysis, we examined the factors of stone clearance (alcohol etiology, stone location, stone number, stone size, and success/failure of guidewire negotiation).

Written informed consent was obtained from each patient prior to performing treatment. The study protocol conformed to the ethical guidelines of the 1975 Declaration of Helsinki and was approved by the institutional review committee of Toho University Omori Medical Center.

### 2.1. Statistical Analysis

Statistical analysis was performed using SPSS for Windows, version 11.0J (SPSS Inc., Chicago, IL). All continuous variables are presented as means ± standard error. A *P* value < 0.05 was considered significant. Comparisons of the outcome variable (stone fragmentation) were analyzed using the Chi-squared test or Fisher's exact test.

## 3. Results

A total of 98 patients with symptomatic chronic pancreatitis underwent endoscopic lithotomy for chronic calcific pancreatitis at our center between May 2005 and December 2012 (Table 1). Patient background and stone factors are given in Table 1.

In total, 89 patients (90.8%) had abdominal symptoms with abdominal pain or discomfort (Table 2(a)). Of the 82 patients with abdominal pain, target pancreatic stones were successfully removed from 64. Although the 17 remaining patients in which stone clearance initially failed were defined as technical failures, they were finally determined to be clinical successes because abdominal pain was improved.

Of the patients in whom abdominal pain was not improved by treatment, 2 underwent successful stone lithotripsy by ESWL at another institution, 1continued pancreatic stent placement, 3 underwent surgery because of continuing pain, and 1 was placed under observation according to the patient's request. Nine patients (9.2%) with no abdominal symptoms and 1 patient defined as a technical failure were followed as outpatients (Table 2(b)).

Stone fragment extraction by combined ET/ESWL therapy was successful in 67 of 98 patients (74.5%), while that by additional EHL was successful in 7 patients (7.1%; 3 cases with the SpyGlass system and 4 cases with direct EHL). Direct EHL was useful in cases when ENPD or EPS placement was unsuccessful. In the 6 (6.1%) cases where EHL was not successful, outpatient ESWL was successful in all 6 cases (Table 3).

Table 4 shows the 31 cases where combined ET/ESWL was not successful. Twelve patients had radiolucent stones, 5 of whom failed to respond to selective pancreatic duct cannulation. One patient subsequently underwent successful outpatient ESWL. Although 15 patients were asymptomatic at this point, they were followed as outpatients, and 2 ultimately required surgery because of no improvement in pain.  Table 5 shows the treatment outcomes of the POPS guided EHL procedures including the SpyGlass Direct Visualization system. Reasons for failure were insufficient dilatation of MPD stricture in 2 patients, direct vision failure in 2 patients, and equipment failure in 1 patient. In addition, perforation by the guidewire occurred in 1 patient. Pancreatitis was improved by conservative treatment in 1 patient (Cotton classification [8]: mild).  Table 6 shows the outcomes of X-ray guided EHL procedures. Yet, 4 of the 6 cases of X-ray guided EHL in this study were treated successfully, and even though the remaining 2 cases were defined as unsuccessful, 1 was successfully treated by ESWL at another institution. However, severe pancreatitis (Cotton classification: severe) due to guidewire perforation occurred in 1 successful case.

Univariate analysis revealed that guidewire negotiation was associated with a significantly higher rate of stone fragmentation than the other methods (*P* = 0.0004) (Table 7). This finding was confirmed by multiple logistic regression analysis of factors in the success group and failure group (*P* = 0.0003) (Table 8).

## 4. Discussion 

The high safety and efficacy of ESWL make it the preferred treatment for patients with painful calcified chronic pancreatitis, and it has been the treatment of choice for clearing pancreatic stones since 1987 [2, 3]. Combined systematic endoscopy with ESWL has been reported to increase the cost of patient care without improving the outcome of pancreatic pain [3]. In our experience, treatment improved abdominal symptoms in 82 of 89 patients. The most ideal treatment is to remove the target stone and dilate the severe stricture of the MPD. However, several cases of severe MPD stricture resulted in a decreased rate of stone clearance. In these cases, EPST or precut reduced MPD hypertension, which in turn reduced abdominal pain.

Cholangioscopy, initially introduced in 1975 using the mother-baby system, has been used to evaluate indeterminate pancreatobiliary diseases. However, the conventional baby scope is a fragile instrument requiring frequent repairs [9, 10]. We chose, therefore, to use the SpyGlass optical probe, which is 0.9 mm in diameter and can be inserted through an endoscopic ERCP catheter [11]. ERCP can, therefore, be performed more easily and quickly than conventional mother-baby cholangiopancreatoscopy. As in cases of pancreatic lithiasis with MPD stricture, ultimate upangulation is required, but this leads to optical probe damage. The SpyGlass system uses a replaceable optical probe and a disposable access catheter, and it has 4-way deflected steering with separate dedicated irrigation channels which obviates the need to send out the cholangioscope for repairs. Furthermore, because the SpyScope is a disposable catheter, which means it is cost effective, it is considered the first choice for pancreatic lithiasis EHL cases [12].

With regard to POPS guided EHL outcomes, Craigie et al. reported the following factors for the mother-baby scope system: (a) stone burden (i.e., near total impaction of concretions in the pancreatic head); (b) acute angulation of the main pancreatic duct at the genu and inability to navigate this junction with a guidewire or scope; and (c) baby scope failure due to the instrument being fragile [6]. Based on these findings, even though the SpyGlass Direct Visualization system has a 4-way angled system, it has limited front-viewing control in severe cases of MPD stricture compared with that in the wider common bile duct [13].

On the other hand, performing X-ray guided EHL in the pancreatic duct carries the risk of duct perforation and bleeding. In our department, X-ray guided EHL is performed by the same highly skilled surgeon, which may be one of the factors contributing to the successful outcomes. However, like in our experience with the successful cases of severe pancreatitis, no previous studies have reported X-ray guided EHL similar to the procedure used at our department, suggesting the efficacy of pancreatic stone lithotripsy.

Although combined ET/ESWL therapy is typically an effective treatment for pancreatic stones >10 mm, patients are limited to a hospital stay of 30 days at our institution. Among our 98 patients, 74 had multiple stones and 62 had MPD stricture. Accordingly, only about 10–15 ESWL sessions could be performed during the hospitalization period. Therefore, when the stone remains after 10 ESWL sessions and treatment is expected to be prolonged, either POPS guided EHL or X-ray guided EHL should be performed. Our findings revealed that, in cases where combined ET/ESWL therapy was not successful, a next attempt at EHL increased the stone fragmentation rate.

Furthermore, in cases of failed EHL treatment, we could continue ESWL on an outpatient basis if there was at least some “space” in the MPD. In these cases, the ENPD or pancreatic stent could not be placed as no treatments are suitable for radiolucent stones in cases with severe MPD stricture. Unfortunately, cases of unresolved abdominal pain require surgical treatment [14, 15].

Previous studies reported that the presence of a downstream stricture and stone size and location influence stone fragmentation and clearance [16, 17]. In our study, based on the results of univariate and multivariate analysis, as shown in Tables 7 and 8, no significant differences were observed between stone clearance and the different etiologies (alcohol, stone location, stone size, and stone number). In fact, complete stone clearance was significantly improved by guidewire negotiation, possibly because the success of guidewire negotiation through the MPD stricture has a greater therapeutic effect during ERCP stone clearance therapy.

There are several limitations to this study. First, it is a retrospective study. Second, the use of POPS guided or X-ray guided EHL was determined from the response to the combined ET/ESWL treatment and was not performed in a randomized fashion.

## 5. Conclusions

In cases where stone clearance was unsuccessful by combined ET/ESWL treatment, EHL using the SpyGlass system or X-ray guided EHL was effective when the guidewire could be negotiated through the MPD stricture. Furthermore, although the SpyGlass system has 4-way tip deflection, it is necessary to pay attention in cases of extremely tortuous and narrow MPD. Further prospective randomized EHL studies are needed to verify our findings.

## Figures and Tables

**Table 1:** Outcomes of ERCP/ESWL based on patient background and stone characteristics.

Factor	Value
Age, years (median, range)	54.8 ± 13 (21–81)
Sex	
Male/female	78/20
Etiology	
Alcohol	77
Divisum	8
Idiopathic	6
Genetic	1
Hyperparathyroidism	1
Other	5
Stone size	—
>15 mm/≤15 mm	11/87
Stone location	
Single (*n* = 24)	
Head	18
Body/tail	6
Multiple (*n* = 74)	
Head	58
Body/tail	16
MPD stricture	
Yes/no	62/36

ESWL: extracorporeal shock wave lithotripsy; MPD: main pancreatic duct.

**Table:** (a) Abdominal symptoms before treatment (*n* = 89, 90.8%)

Clinical success	*n*	Clinical failure (no pain improvement)	*n*
Technical success	64	Technical success	0
Technical failure (outcomes)		Technical failure outcome (outcomes)	
Surgery	0	Surgery	3
Outpatient ESWL	1	ESWL at other institutions	2
Pancreatic stent placement	0	Pancreatic stent placement	1
Observation	17	Observation	1

	82 (83.7%)		7 (7.1%)

**Table:** (b) No abdominal symptoms before treatment (*n* = 9, 9.2%)

	*n*
Technical success	8
Technical failure (outcomes)	
Observation	1

	9 (9.2%)

**Table 3:** Outcomes of pancreatic stone treatments.

	Successful ENPD placement or EPST		Total	(%)
	Yes (%)	No (%)		
Combined ET/ESWL therapy success	50	17	67	74.5%
EHL success (POPS EHL/X-ray EHL)	4 (3/1)	3 (0/3)	7 (3/4)	7.1%
Outpatient ESWL success	2	4	6	6.1%

	56 (57.1)	24 (24.5)	80	81.6%

ENPD: endoscopic nasopancreatic drain; EPST: endoscopic pancreatic sphincterotomy; ET: endoscopic treatment; ESWL: extracorporeal shock wave lithotripsy; EHL: electrohydraulic lithotripsy; PPS: prophylactic pancreatic stent.

**Table 4:** Failure cases of combined ET/ESWL treatment (*n* = 31).

	Successful ENPD placement or EPST		Total success (%)
	Yes	No	
PPS EHL (*n* = 8)			
Success	3	—	3
Failure			
ESWL (success)	1	3	4
Followup	—	1	
Direct EHL (*n* = 6)			
Success	1	3	4
Failure			
ESWL (success)	1	—	1
Followup	1	—	
Radiolucent stones (*n* = 12)			
Followup	2	9	
Surgery	—	1	
EPST or precut failure (*n* = 5)			
ESWL success	—	1	1
Followup	—	3	
Surgery	—	1	

Total success cases	6/9	7/22	13/31 (41.9)

ENPD: endoscopic nasopancreatic drain; EPST: endoscopic pancreatic sphincterotomy; ET/ESWL: combined endoscopic treatment/extracorporeal shock wave lithotripsy; EHL: electrohydraulic lithotripsy; PPS: prophylactic pancreatic stent.

**Table 5:** Outcomes of peroral pancreatography-guided EHL procedures.

Number	Location	Stone diameter (mm)	Number of stones	Reason for failure	ENPD placement/EPST	Complication	Outcome
1.	Head	13	Multiple	—	Success	—	
2.	Head	12	Multiple	—	Success	—	
3.	Head	14	Single	—	Success	—	
4.*	Head	20	Diffuse	Severe stricture	Failure	—	Other institution ESWL
5.	Head	10	Single	Severe stricture	Failure	—	Outpatient ES
6.	Head	10	Multiple	Direct vision failure	Failure	Perforation	Observation
7.	Head	12	Single	Direct vision failure	Failure	Pancreatitis	Observation
8.	Head	10	Single	Equipment failure	Failure	—	Combined ET/ESWL

*CHF BP 260 was used in case 4 for EHL.

**Table 6:** Outcomes of X-ray guided EHL procedures.

Number	Location	Stone diameter (mm)	Number of stones	Reason for failure	ENPD placement/EPST	Complication	Outcome
1.	Head	8	Multiple	—	Success	—	
2.	Body	10	Multiple	—	Success	—	
3.	Head	16	Single	—	Success	Pancreatitis, GW perforation, and pancreatic abscess	Discharge
4.	Head	9	Multiple	—	Success	—	
5.	Body	7	Multiple	Severe stricture	Failure	—	Other institution ESWL
6.	Body	10	Multiple	Severe stricture	Failure	Pancreatitis	Observation

**Table 7:** Univariate analysis of stone clearance.

	Success group (*n* = 74)	Failure group (*n* = 24)	OR (95% CI)	*P*
Alcohol etiology (yes/no)	60/14	19/5	1.13 (0.26–5.05)	0.87
Stone location (head/body or tail)	57/17	18/6	3.46 (0.80–14.87)	0.09
Stone number (single/multiple)	21/53	6/18	0.69 (0.16–2.88)	0.61
Guidewire negotiation (success/failure)	68/6	9/15	32.1 (7.67–134.97)	0.0004
Stone size (≤15 mm/>15 mm)	75/9	12/2	0.89 (0.77–1.03)	0.13

OR: odds ratio; CI: confidence interval.

**Table 8:** Multivariate analysis of stone clearance.

	Success group	Failure group	OR (95% CI)	*P*
Guidewire negotiation (success/failure)	68/6	9/15	14.1 (0.46–43.21)	0.0003

OR: odds ratio; CI: confidence interval.
